# Species-Resolved Metagenomics of Kindergarten Microbiomes Reveal Microbial Admixture Within Sites and Potential Microbial Hazards

**DOI:** 10.3389/fmicb.2022.871017

**Published:** 2022-03-28

**Authors:** TzeHau Lam, Dillon Chew, Helen Zhao, Pengfei Zhu, Lili Zhang, Yajie Dai, Jiquan Liu, Jian Xu

**Affiliations:** ^1^Global BioScience, Procter & Gamble Singapore Innovation Center, Singapore, Singapore; ^2^Single-Cell Center, CAS Key Laboratory of Biofuels and Shandong Key Laboratory of Energy Genetics, Shandong Energy Institute, Qingdao Institute of BioEnergy and Bioprocess Technology, Chinese Academy of Sciences, Qingdao, China; ^3^College of Life Science, University of Chinese Academy of Sciences, Beijing, China

**Keywords:** kindergarten, environmental surfaces, microbiome, metagenomics, microbial admixture

## Abstract

Microbiomes on surfaces in kindergartens, the intermediate transfer medium for microbial exchange, can exert significant impact on the hygiene and wellbeing of young children, both individually and as a community. Here employing 2bRAD-M, a novel species-resolved metagenomics approach for low-biomass microbiomes, we surveyed over 100 samples from seven frequently contacted surfaces by children, plus individual children’s palms, in two kindergartens. Microbiome compositions, although kindergarten-specific, were grouped closely based on the type of surface within each kindergarten. Extensive microbial admixture were found among the various sampled sites, likely facilitated by contact with children’s hands. Notably, bacterial species with potential human health concerns and potentially antibiotic-resistant, although found across all sampled locations, were predominantly enriched on children’s hands instead of on the environmental sites. This first species-resolved kindergarten microbiome survey underscores the importance of good hand hygiene practices in kindergartens and provides insights into better managing hygiene levels and minimizing spread of harmful microbes in susceptible indoor environments.

## Introduction

Modern human beings spend most of their life time indoors. They are surrounded by indoor microbiomes, which can exert significant impact on their short-term and long-term health. For example, young children typically spend up to 8 h daily at the kindergarten. Aggravated by the high children density and the lack of proper hygiene habits in children, the kindergarten environment can be exposed to a sizeable diversity of pathogenic microorganisms (Bartlett et al., 1985; Lee et al., 2007), and thus become perfect hotspots for disease transmission (Venczel et al., 2001; Lu et al., 2004; Marziano et al., 2018). In fact, young children prevail as the most vulnerable group to infectious agents, as evidenced by their higher infection, hospital admission, and mortality rates than other age groups (Li-Kim-Moy et al., 2017; Dong et al., 2020). Therefore, knowledge of the kindergarten microbiome can provide a better understanding toward microbial hotspots, which can guide optimal crowd hygiene behavior and infection control practices for better protecting children’s health.

The prevalence of common microorganisms in the kindergarten setting has been well documented (Liu et al., 2000; Ibfelt, 2015; Ibfelt et al., 2015). However, past studies often employed culture-based methods or molecular techniques, such as quantitative polymerase chain reaction which targets only a limited number of microorganisms, thereby are incapable of detecting all potential microbes (Chiu and Miller, 2019). In addition, 16S rRNA gene amplicon sequencing used to survey surface contamination in childcare facilities lacks the required taxonomic resolution to accurately characterize microbes at the species level (Lee et al., 2007; Shin et al., 2015). In view of the above limitations, metagenomic next-generation sequencing approaches are attractive alternatives as they enable comprehensive and unbiased taxonomic characterization of the microbiome at species-level resolution (Chiu and Miller, 2019; Gu et al., 2019).

Unlike human microbiomes, samples collected from the built environment are often of low biomass and can be heavily degraded, thus applying metagenomic sequencing techniques on such samples has been difficult (Horve et al., 2020). To tackle such challenges, we have recently established a new metagenome reduction sequencing approach called 2bRAD-M and showed that it is particularly advantageous for profiling low-biomass, degraded or contaminated microbiomes at the “species” resolution (Sun et al., 2022). Therefore, employing 2bRAD-M and using kindergarten as a model for indoor environment, here we profiled the surface-associated microbiomes at the species resolution and probed possible children–environment–microbe interactions.

## Materials and Methods

### Sample Collection and Processing

Samples were taken inside typical kindergartens located within the Shinan (Kindergarten 1) and the Laoshan (Kindergarten 2) residential district in Qingdao, Shandong, China. Sampling for both kindergartens was conducted on the morning and afternoon of January 3, 2020 in a single session. No conditions or restrictions in terms of the surface cleaning frequency or the children’s daily activities were imposed prior to the sampling day. A total of 99 samples from seven indoor sites were collected simultaneously, including Playroom Floor (four samples in kindergarten 1; five samples in kindergarten 2), Toilet Floor (five samples each), Color Pencil (10 samples each), Playroom Dust (five samples each), School Bag (five samples each), Picture Book (10 samples each), and Toy Surface (10 samples each). In addition, samples were taken from the palms of fifteen 3- to 6-year-old children before handwashing in kindergarten 2.

Sterile flocked swabs were moistened with sterile deionized water (0.15 M NaCl & 0.1% Tween 20) and then applied transversely and longitudinally for 20 times within an 8 cm × 8 cm area. For color pencils and toy surfaces, the swabs were wiped to cover the entire surface. For each child, the whole palm of both hands was swiped by a swab before handwashing. The collected swabs were then transferred into 2-ml Eppendorf tubes containing 500 μl sterile RNase-free buffer and stored at −80°C prior to DNA extraction. Playroom floor dust was collected by a vacuum cleaner (Panasonic, MC-WF550, 700 W) attached to a Dustream® collector (Indoor Biotechnologies, Charlottesville, United States). Dust collection was conducted for 2 min along the edge of the playroom floor for each sampling site. After sampling, the filter column was removed from the collector, sealed, and stored in Ziploc bags at −80°C prior to DNA extraction.

### Genomic DNA Extraction, Library Preparation, and Metagenomic Sequencing

Genomic DNA was extracted using TIANamp Micro DNA Kit (Tiangen, Beijing, China). Carrier RNA was added to increase the yield, and extracted DNA was eluted in the 20 μl RNase-free water. After DNA extraction, 2bRAD libraries were prepared according to Sun et al. (2022). Briefly, 4 U of BcgI (NEB, United States) restriction enzyme was used to digest the genomic DNA at 37°C for 3 h. Next, ligation reaction with 0.2 μM of library-specific adaptors (Ada1, Ada2) at 4°C for 16 h was performed before heat inactivation at 65°C for 20 min for BcgI. Polymerase chain reaction (PCR) was carried out on the ligation products by including 7 μl ligated DNA, 0.1 μM primers, 0.3 mM dNTP, 1× Phusion HF buffer, and 0.4 U Phusion High Fidelity DNA polymerase (NEB, United States) at the following conditions—16–28 cycles at 98°C for 5 s, 60°C for 20 s, 72°C for 10 s, and final extension of 10 min at 72°C. The resulting libraries were purified with the QIAquick PCR purification kit (Qiagen, Valencia, CA) and sequenced on Illumina HiSeq X™ Ten platform. Library construction and sequencing were performed in OE BioTech Co., Ltd., Qingdao.

### Sequence Processing and Analysis

Raw sequences were processed and selected using the fastp sequence QC tool (Chen et al., 2018) with the following criteria: (i) read length of 32 bp and (ii) Phred quality score of bp >25. This yielded high-quality BcgI enzyme-digested sequence fragments of 32 bp, yielding a total of 279 million reads with an average of 2.48 million reads per sample. Taxonomic profiles were determined using the 2bRAD-M computational pipeline; GitHub: https://github.com/shihuang047/2bRAD-M (Sun et al., 2022). Briefly, reads were mapped against a pre-determined 2bRAD tag database which contains taxa-specific BcgI-derived sequences identified from 173,165 RefSeq microbial genomes. Reads coverage for each identified genome was determined and used to estimate the relative abundance of each taxon. To ensure reliable taxonomic profiling, identified taxonomic units not meeting the following criteria were discarded from downstream analysis: (i) taxonomy with taxa-specific 2bRAD-tag number < 5, and (ii) taxonomy with sequenced reads number < 15. Eventually, 495 microbial species across the 114 samples remained in our datasets for further analysis.

To check against potential cross-contamination from reagents during preparation, the ATCC® MSA-1002™ mock microbial community consisting of 20 bacteria species at even abundance of genomic DNA at ~5 ng was prepared as a positive control. Library construction & sequencing of the control were performed in conjunction with the rest of the samples. Detail taxonomic analysis revealed the detection of the 20 bacteria species at approximate equal relative abundance that is consistent with the expected profile (Supplementary Table 1). In addition, the principal coordinates analysis (PCoA) plot showed that taxonomic profile of the mock control is distinct from most of the samples (Supplementary Figure 1), suggesting minimal cross-contamination.

### Derivation of Ecological Characteristics for Microbiomes

The R package Vegan v2.5.7 was used to calculate Shannon diversity and perform PCoA ordination (with Bray–Curtis dissimilarity as the distance measure) based on the taxonomic abundance profiles of each sample in our dataset. Difference in community structures was evaluated by PERMANOVA test conducted with 1,000 permutations. All other statistical tests were carried out with the Stats v4.0.2 and Vegan v2.5.7 R packages. SourceTracker2 (McGhee et al., 2020) version 2.0.1 was used to estimate the proportion of microbial contribution at each sampled site that comes from the other sampled sites in the same kindergarten. Identification of co-occurring samples and species was performed using SpiecEasi R package version 1.1.1 (Kurtz et al., 2015). Network graph inference was built using all species of mean relative abundance of >0.001 and with Meinshausen and Buhlmann’s neighborhood selection. All graphical plots were generated *via* the R packages of ggplot2, ggpubr, and ggnet2.

## Results

### Microbial Communities Differ Between the Two Kindergartens and Among Surface Sites

**Figure 1:**
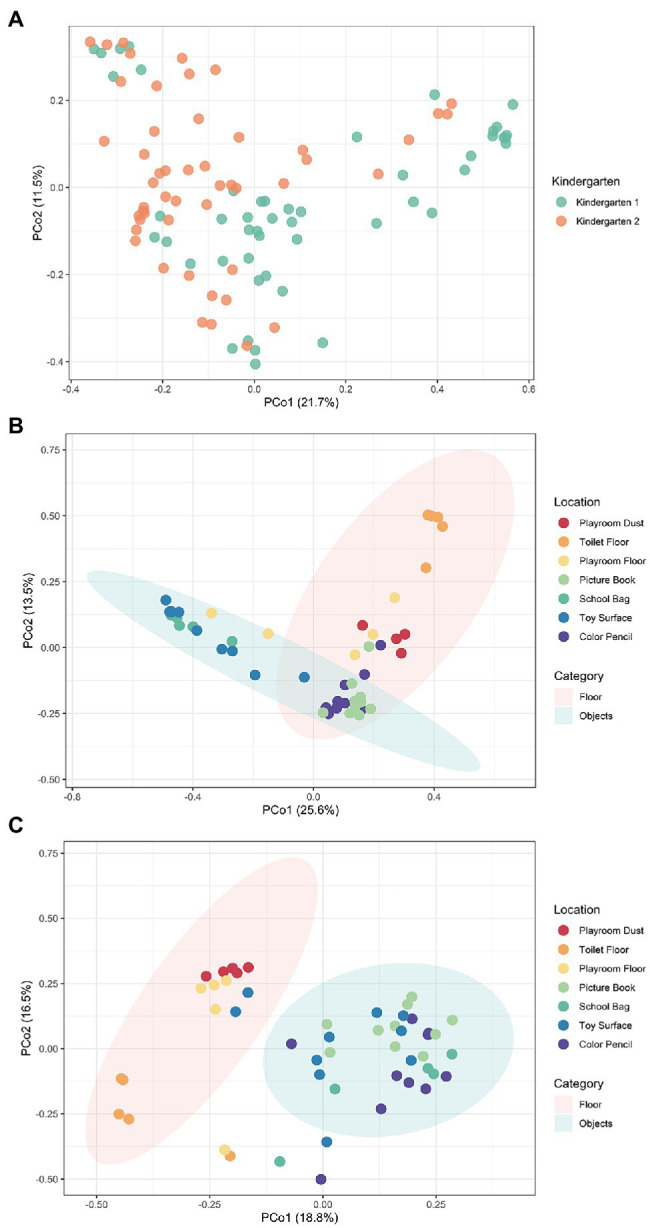
The species-level microbial compositional profiles of the kindergartens visualized using the principal coordinate analysis (PCoA) based on Bray–Curtis distances. **(A)** The PCoA together for both kindergartens revealed significantly different compositions of microbial species (PERMANOVA; *R*^2^ = 0.059, *p* < 0.001) between the two kindergartens. Moreover, individual PCoA was conducted separately for both **(B)** Kindergarten 1 and **(C)** Kindergarten 2, revealing significantly different composition of microbial species (kindergarten 1: PERMANOVA; *R^2^* = 0.467, *p* < 0.001, kindergarten 2: PERMANOVA; *R^2^* = 0.366, *p* < 0.001) between samples collected from the floor (Playroom Dust, Playroom Floor, and Toilet Floor), and those collected from hand-touched objects (Picture Book, School Bag, Toy Surface, and Color Pencil). Each point corresponds to a sample and is colored according to the kindergarten or sampling site. Ellipses correspond to 95% confidence regions.

We started by collecting 99 indoor surface samples from seven non-host-body sites frequented by children (Playroom Floor, Toilet Floor, Playroom Dust, Color Pencil, School Bag, Toy Surface, and Picture Book) in both kindergartens and the microbiomes were sequenced (Section “Materials and Methods”). The Bacteria kingdom accounts for more than 95% of relative abundance across all samples. Fungi were mostly concentrated in the Playroom Dust and Playroom Floor, with mainly Ascomycota and Basidiomycota found (Supplementary Figure 2). At the species level, the predominant taxa shared across the sites were primarily the Gram-negative bacteria of *Moraxella osloensis* (Kindergarten 1: 6.1%, Kindergarten 2: 6.7%) and *Acinetobacter johnsonii* (Kindergarten 1: 3.9%, Kindergarten 2: 9.6%; Supplementary Figure 3), which were reported in past studies of daycare facilities (Andersson et al., 1999; Lee et al., 2007; Shin et al., 2015). However, the distinction between the two kindergartens becomes more obvious from a community standpoint, where PCoA with Bray–Curtis dissimilarity illustrates significant difference (permutational multivariate analysis of variance, PERMANOVA; *R^2^* = 0.059, *p* < 0.001) in microbial compositions between the two kindergartens (Figure 1A). Compositional differences between the two kindergartens were more distinct at sites such as the Toilet Floor and Toy Surface where samples obtained from the same site in one kindergarten clustered far away from those in the other kindergarten (Supplementary Figure 1). This observation is further supported by differences in the species-level alpha diversity, where the Shannon diversity index was observed to be significantly different between the two kindergartens at the Toilet Floor (*p* = 0.016) and the Toy Surface (*p* = 0.015; Supplementary Figure 4). Conversely, Playroom Dust samples from both kindergartens were observed to cluster closely (Supplementary Figure 1), suggesting a certain level of dust microbiome homogeneity possibly due to similar levels of urbanization and environmental conditions between the districts where the two kindergartens were located (Shan et al., 2020; Park et al., 2021).

Besides the differences between kindergartens, the microbial profiles within each kindergarten were also observed to group closely based on the type of surface where the samples were collected from. In both kindergartens, samples collected from the floor (Playroom Dust, Toilet Floor, and Playroom Floor) were observed to form a distinctive cluster that is separate from samples collected from those hand-touched objects (School Bag, Toy Surface, Picture Book, and Color Pencil), suggesting that the microbiomes between these two types of surfaces are distinct (Figures 1B,C). Not surprisingly, dominant species found on hand-touched objects across both kindergartens were largely associated with the human microbiota (e.g., *Cutibacterium acnes* and *Streptococcus* spp.), while dominant species found on the floor (e.g., *Paracoccus* spp. and *Pseudomonas* spp.) were commonly associated with the environment (Supplementary Figure 3). In particular, within each site, samples collected from the floor were relatively conserved (i.e., they cluster together based on the originated site), while those collected from objects were more diverse. Together, these observations suggest that the microbial communities of each site are heavily shaped by interaction with the environment and by human behaviors.

### Admixture of Microbes Between Sampled Surfaces

**Figure 2:**
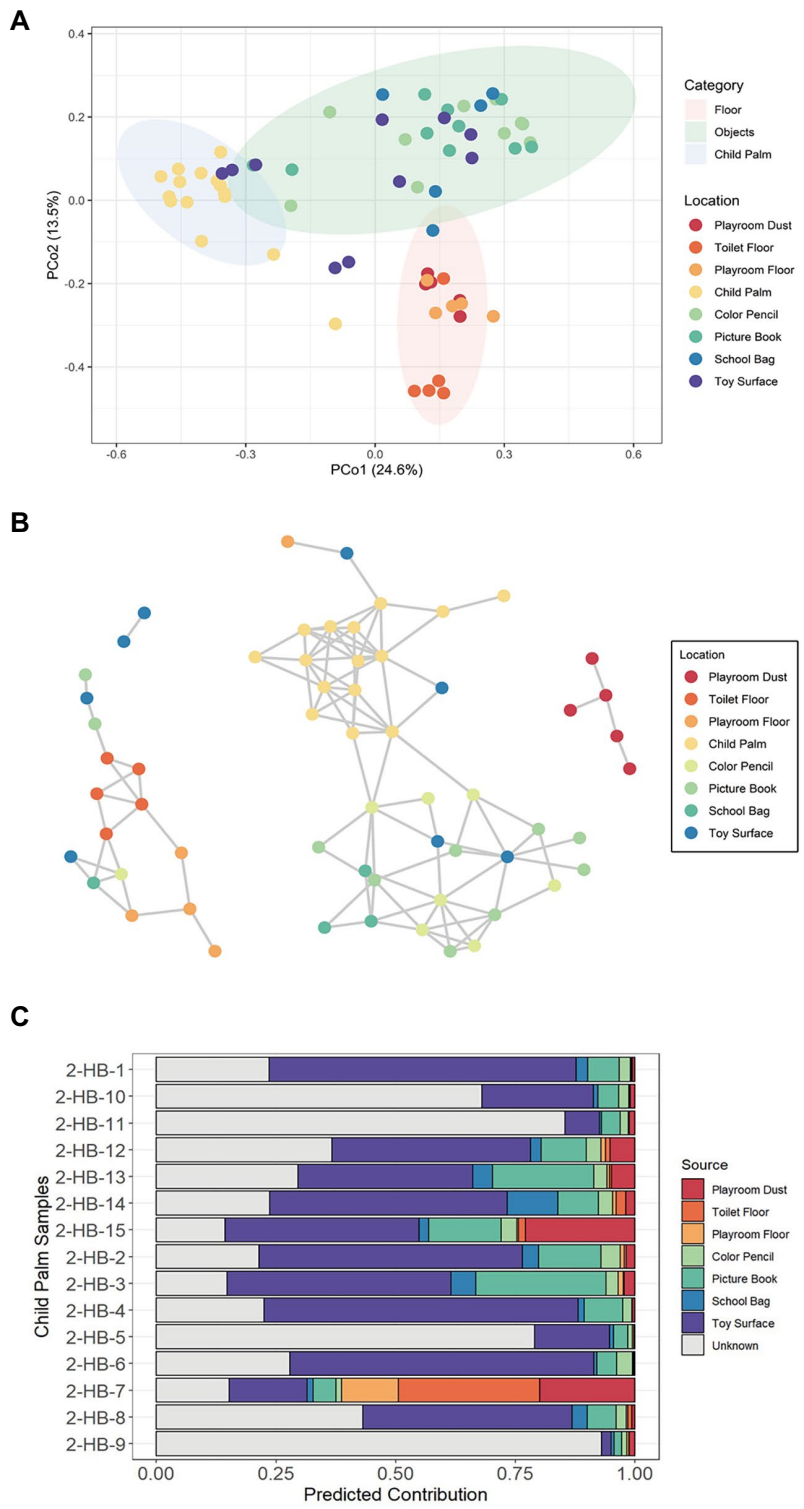
Microbial associations between sampled sites with the inclusion of child palm samples. **(A)** Principal coordinates analysis (PCoA) ordination plot based on Bray–Curtis distance reveals significantly different composition of microbial species (PERMANOVA; *R^2^* = 0.410, *p* < 0.001) among the sampled sites of Kindergarten 2. Three distinct clusters corresponding to samples obtained from the floor (Playroom Dust, Playroom Floor, and Toilet Floor), hand-touched objects (Picture Book, School Bag, Toy Surface, and Color Pencil), and the child palm were formed. Each point corresponds to a sample and is colored according to the sampled site. Ellipses correspond to 95% confidence regions. **(B)** Inference of microbial interaction network based on inter-sample correlation in Kindergarten 2 using SPIEC-EASI based on the relative abundance of all bacterial species. Sample-to-sample interactions were stratified by the sampling site. Each node represents a sample with the color indicating the sampling site, and no samples were omitted from the network. **(C)** The relative contribution from each microbial “source” community to each child palm (“sink”) microbial community was estimated using SourceTracker.

Despite the among-site distinctiveness of surface microbiomes, microbial exchange between surfaces is expected to be common (Meadow et al., 2014; Stephens et al., 2019). While most microbial taxa are likely to perish over time, some can lay dormant or even survive to co-inhabit with the native microbial populations (Stephens et al., 2019). Noting that hands are the most common vehicle for the transmission of microorganisms between children’s body and their environmental surfaces (Lau et al., 2012), we sought to pinpoint the potential sources of the microbes present on the child palm as well as exchanges with the kindergarten surfaces. Although a significant difference in alpha diversity was not observed on child palm as compared to the other sampled locations except the Color Pencil (Supplementary Figure 5A), PcoA analysis revealed that the child palm samples exhibit distinctive distribution of microbial composition as compared to those collected from the floor and objects (Figure 2A). This reflects on the specific ecological niche to sustain a distinct microbial community, with dominant species identified being common skin flora such as *Cutibacterium acnes* (Supplementary Figure 5B). Interestingly, we also observed an abundance of species associated with the oral microbiome such as *Streptococcus mitis*, *Streptococcus oralis*, and *Streptococcus sanguinis* on the child palm (Supplementary Figure 5B), possibly due to the increased hand-to-mouth habits of young children such as nail biting and/or finger sucking (Tulve et al., 2002). Adults on the other hand, as shown in a previous study, have a lower relative abundance of Streptococcaceae as compared to infants and children (Song et al., 2013). Additionally, we also observed overlapping of toy surface and picture book samples with the child palm cluster, suggesting similar microbial compositions between these samples.

To gain insight into the possibility of children–microbe interactions across surfaces, we infer the microbial interaction network among all the sampled surfaces based on the relative abundance of all identified species. In the network, three main co-occurrence clusters were identified, namely the Toilet Floor/Playroom Floor cluster, the Child Palm/Objects cluster, and the Playroom Dust cluster (the final cluster solely consists of dust samples collected from the playroom; Figure 2B). Interestingly, species co-occurrences (as denoted by edges) between child palm samples and samples from Toy Surface and Color Pencil were observed, suggesting the inside-kindergarten cross-transmission routes as mediated by children’s hands. In addition, the observation of interconnections between samples from Color Pencil, Picture Book, School Bag, and Toy Surface suggest admixture of bacterial species, which is possibly facilitated by contact instances with children’s hands. The disconnection between the Child Palm/Objects cluster and the other two clusters can be potentially explained by lower/shorter contact or non-contact instances of the floor surfaces with the children’s hands.

Building on this observation, we then investigated the potential sources of the microbes present on child palm as well as its cross-transmission with the kindergarten surfaces. Using the Bayesian approach (Knights et al., 2011) to estimate proportion of the given “sink” samples (child palm) that are comprised of species from a potential “source” community (i.e., a surface), we found that Toy Surface is the dominant “source” with a predicted mean contribution of 33.68% across all samples (Figure 2C), followed by Picture Book (10.81%). This result supports the intuitive notion of commonly touched objects representing the primary source of bacteria found on hands, where the two locations are common contact hotspots most likely having serving as potential intermediaries for cross-transmission to/from children’s palms.

### Prevalence of Potential Microbial Hazards in Kindergartens

**Figure 3:**
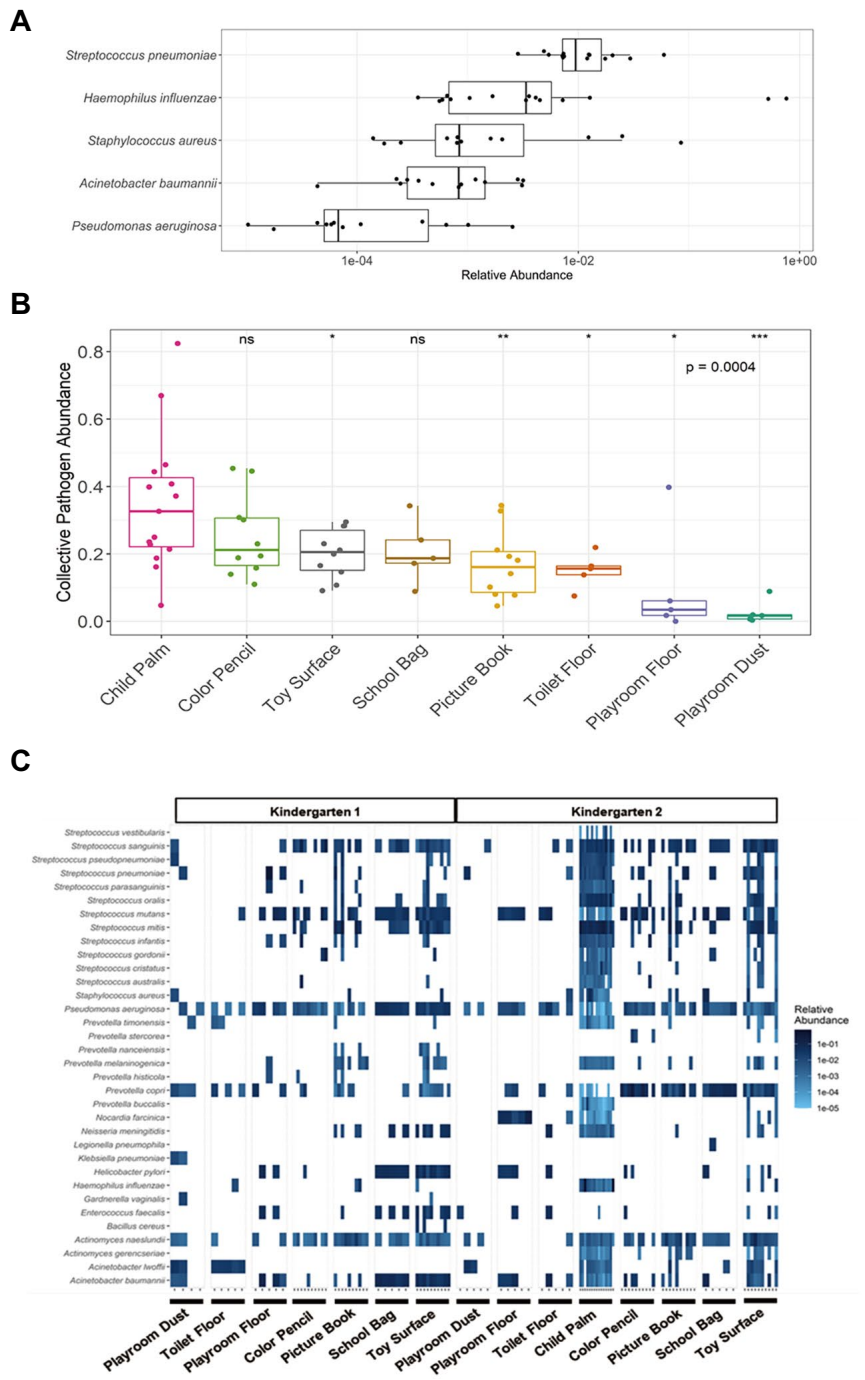
Presence of potential microbial hazards in various surfaces of kindergartens. **(A)** Distribution of potentially antibiotic-resistant bacteria on Child Palm (*n* = 15) with mean relative abundance of >0.001%. **(B)** Significant difference in collective relative abundance of identified microbes with potential human health concerns was observed across the seven sampled locations and child palm in Kindergarten 2(*p* = 4.18 × 10^−4^, Kruskal–Wallis). With Child Palm as the reference group, Toy Surface (*p* = 0.0357), Picture Book (*p* = 0.0080), Toilet Floor (*p* = 0.0107), Playroom Floor (*p* = 0.0146) and Playroom Dust (*p* = 2.58 × 10^−4^) showed significantly lower relative abundance of pathogens. The *p* values above the boxplots were derived via the Mann–Whitney U-test: n.s., not significant, *p ≤ 0.05, **p ≤ 0.01, ***p ≤ 0.001. **(C)** Heatmap of the identified microbes of potential human health concern across the seven different sampled sites of both kindergartens and the child palms. Only those species with mean relative abundance of >0.001% across the individual sites were included.

Elucidation of the microbial communities at the species resolution offers an opportunity for a comprehensive understanding of the relative microbial hazards among the surfaces within the kindergarten setting. Such hazards include potentially antibiotic-resistant bacteria and microbes which are of potential human health concerns. For example, the World Health Organization published a list of “priority bacterial pathogens” with substantial risk of antibiotic resistance that encompass a catalogue of 12 pathogenic family/genus/species which can cause food poisoning, common diseases, and severe infections (World Health Organization, 2017). Across the sampled locations from both kindergartens, a total of eight such bacteria species were detected at the species level, with at least one species detected in 74 samples, yielding an overall detection rate of 88.1% (species with mean relative abundance of >0.001%). Among the species, *Pseudomonas aeruginosa* was the most prevalent with a detection rate of 98.8%, followed by *Acinetobacter baumannii* at 52.4% (Table 1). Although the child palm was observed to harbor five out of the eight identified bacterial species with a detection rate of >80%, the relative abundance of these species was mostly low <1% (Figure 3A). In addition, we observed wide variations in the relative abundance of those potentially antibiotic-resistant bacteria as well as the microbiome among the children palms (Figures 2C, 3A). These can be attributed to the difference in the behaviors of individual child as recent studies had demonstrated that human behaviors and daily routines have a major role in spread of bacterial contaminants and fluctuation of microbiome in the indoor environments (Wang et al., 2021; Wilkins et al., 2021). Between the surfaces, the proportion of toys detected with potentially antibiotic-resistant bacteria (averagely 41.2%) is twice as much when compared to the other surfaces (averagely 20.9%), suggesting toys tend to be overlooked, which should be addressed with frequent hygiene intervention.

**Table 1:** Detection rate of potentially antibiotic-resistant bacteria across all sampled sites in both kindergartens.

Pathogen	Kindergarten 1							Kindergarten 2							
	PF (*n* = 5)	PD (*n* = 4)	TF (*n* = 5)	CP (*n* = 10)	PB (*n* = 10)	SB (*n* = 5)	TS (*n* = 10)	PF (*n* = 5)	PD (*n* = 5)	TF (*n* = 5)	CP (*n* = 10)	PB (*n* = 10)	SB (*n* = 5)	TS (*n* = 10)	Palm (*n* = 15)
*Acinetobacter baumannii*	3 (60%)	2 (50%)	0 (0%)	1 (10%)	3 (30%)	5 (100%)	10 (100%)	2 (40%)	0 (0%)	3 (60%)	4 (40%)	3 (30%)	1 (20%)	7 (70%)	13 (87%)
*Campylobacter* spp.	0 (0%)	1 (25%)	0 (0%)	0 (0%)	0 (0%)	0 (0%)	0 (0%)	0 (0%)	0 (0%)	0 (0%)	0 (0%)	0 (0%)	0 (0%)	0 (0%)	0 (0%)
*Haemophilus influenzae*	0 (0%)	0 (0%)	1 (20%)	0 (0%)	2 (20%)	0 (0%)	1 (10%)	0 (0%)	0 (0%)	0 (0%)	0 (0%)	0 (0%)	0 (0%)	3 (30%)	15 (100%)
*Helicobacter pylori*	2 (40%)	0 (0%)	0 (0%)	1 (10%)	0 (0%)	5 (100%)	10 (100%)	1 (20%)	0 (0%)	3 (60%)	2 (20%)	0 (0%)	1 (20%)	3 (30%)	0 (0%)
*Klebsiella* spp.	0 (0%)	2 (50%)	0 (0%)	0 (0%)	0 (0%)	0 (0%)	0 (0%)	0 (0%)	0 (0%)	0 (0%)	0 (0%)	0 (0%)	0 (0%)	0 (0%)	0 (0%)
*Pseudomonas aeruginosa*	4 (80%)	2 (50%)	3 (60%)	10 (100%)	7 (70%)	5 (100%)	10 (100%)	4 (80%)	2 (40%)	4 (80%)	9 (90%)	8 (80%)	5 (100%)	10 (100%)	12 (80%)
*Staphylococcus aureus*	0 (0%)	1 (25%)	0 (0%)	0 (0%)	1 (10%)	0 (0%)	1 (10%)	1 (20%)	0 (0%)	0 (0%)	0 (0%)	2 (20%)	1 (20%)	2 (20%)	12 (80%)
*Streptococcus pneumoniae*	2 (40%)	1 (25%)	0 (0%)	0 (0%)	3 (30%)	0 (0%)	2 (20%)	1 (20%)	2 (40%)	0 (0%)	3 (30%)	3 (30%)	1 (20%)	7 (70%)	14 (93%)

*The species listed were based on the WHO priority pathogen list (World Health Organization, 2017), with detection defined as having a relative abundance of >0.001%. PF, Playroom Floor; PD, Playroom Dust; TF, Toilet Floor; CP, Color Pencil; PB, Picture Book; SB, School Bag; TS, Toy Surface; Palm, Child Palm*.

To identify potential microbes of human health concern, a total of 299 opportunistic pathogenic bacterial species published by the Chinese Center for Disease Control and Prevention was used as a reference (Miao et al., 2017). A total of 34 distinct potentially pathogenic bacterial species (each with mean relative abundance >0.001%) were identified across all samples with dominant genera (>90% of all identified species) being *Acinetobacter*, *Actinomyces, Prevotella, Pseudomonas*, and *Streptococcus* (Figure 3B). In particular, Child Palm samples were found to carry the highest number of pathogenic species, while floor samples harbor the least. Notably, these species have been observed in previous studies of childcare and daycare centers (Ibfelt et al., 2015; Nygaard and Charnock, 2018), and are mostly associated with respiratory infections (e.g., *Streptococcus pneumoniae* and *Haemophilus influenzae*), gastro-intestinal infections (e.g., *Helicobacter pylori* and *Enterococcus faecalis*), and oral health concerns (e.g., *Actinomyces naeslundii*, *Streptococcus mutans*, and *Streptococcus oralis*). To provide a quantitative view of the potentially pathogenic bacterial species among the surfaces, the collective pathogen abundance was calculated by the summation of the individual species’ relative abundance of the 34 identified potentially pathogenic bacterial species. Notably, a significant difference was observed (*p* = 4.18 × 10^−4^, Kruskal–Wallis) across the different sites in kindergarten 2 (Figure 3C), with Toy Surface (*p* = 0.0357), Picture Book (*p* = 0.0080), Toilet Floor (*p* = 0.0107), Playroom Floor (*p* = 0.0146) and Playroom Dust (*p* = 2.58 × 10^−4^) showing significantly lower collective pathogen abundance than child palm (collective pathogen abundance: 0.349 ± 0.201). Overall, this suggests that children’s hand can possibly harbor more potentially pathogenic bacterial species than those surfaces conventionally considered “dirty” surfaces such as Toilet Floor, and highlights those surfaces where higher standards of sanitation are needed for proper hygiene control in the kindergartens.

## Discussion

In contrast to prior studies that have investigated environmental surfaces in kindergartens *via* culture-based methods or molecular techniques, this study is the first species-resolved metagenomic characterization of microbial communities present on surfaces frequented by child activities at kindergartens. The use of 2bRAD-M, a metagenome reduction sequencing approach, allowed for the profiling of low-biomass microbiomes, revealing not just the prevalence of pathogens but also the possible microbial flow and interactions among surfaces in the kindergarten. Collectively, these results confirm and deepen the findings from several previous studies which revealed the diversity of microorganisms in the kindergarten environment, microbial contact hotspots, and prevalence of potential pathogens (Lee et al., 2007; Ibfelt, 2015; Ibfelt et al., 2015; Nygaard and Charnock, 2018).

Interestingly, despite being in neighboring districts of the same city and thus presumably subjected to similar environmental factors such as relative humidity and temperature, the two kindergartens exhibit clear differences in their microbiomes. Thus, it is probable that occupational factors such as number of occupants, length of occupant stay, and occupant behavior can have a larger influence in shaping the microbiome, supporting existing findings that the building environmental conditions often have a small influence on indoor bacterial communities (Barberán et al., 2015; Stephens, 2016).

Although our analysis infers the admixture of microbes across the kindergarten surfaces which was possibly facilitated through hand contact, the detection of microbes on kindergarten surfaces does not assert their source, nor establish that they can be further transmitted from one surface to another. Surface-to-surface transmission of microbes depends on the characteristics of the microbes and surface itself and would require in-depth complementary laboratory investigations to determine if the detected microbes are indeed deposited through human contact. Nonetheless, our findings indicate that commonly touched objects are potential hotspots which can facilitate the exchange of microbes during direct hand contact, thus increasing the children’s risk of exposure to potential microbial hazards. In particular, *S. pneumoniae*, which was identified in 93% of all child palms in this study, have been demonstrated to spread through direct physical contact and lead to acquisition of nasopharyngeal colonization, potentially increasing the possibility of infection (Connor et al., 2018).

It is also important to note that the detection of DNA signatures arising from bacteria with pathogenic potential in our study does not equate to a higher risk of infection or disease susceptibility as these data do not provide definitive information about their strain type, viability, or pathogenicity. For instance, the likelihood of infection largely depends on the quantity of pathogen exposure, virulence of the pathogen, and whether the pathogen is metabolically active, information which might be challenging to obtain from such sequence-based metagenomic DNA data. Likewise, 2bRAD-M does not provide information about the presence of antimicrobial resistance genes, thus the detection of potentially antibiotic-resistant pathogens only provides an indication of exposure risk. Future studies based on microbial single-cell technologies instead, such as ramanome and Raman-activated Cell Sorting and Sequencing (RACS-Seq), can potentially assess the risks regarding strain type, viability, vitality, and antibiotic resistance (both phenotype and the underlying genotype; Xu et al., 2020; Jing et al., 2021) at the level of individual bacterial cells. Moreover, it should be noted that many of the detected taxa with potential implications for human health were also opportunistic pathogens such as *Actinomyces naeslundii* and *Streptococcus mitis*, which can lead to infections in oral lesions and immune-compromised patients, respectively, but also asymptomatically colonize the human body (Mitchell, 2011; Könönen and Wade, 2015). Furthermore, the values reported in this study are relative abundances, thus it is not yet clear whether a higher relative abundance between kindergartens or sampling locations is due to an increase in a certain potentially pathogenic species, or a decrease in others.

In summary, this study was the first to adopt the 2bRAD-M technology, which overcomes the low-biomass microbiome challenge, for indoor environmental samples to establish an unbiased map of microbiomes within kindergartens and microbial transmissions among indoor sites and the human inhabitants. This enabled the inference of bacterial species admixture across the kindergarten surfaces, possibly facilitated by contact instances with children’s hands. The predominant enrichment of bacterial species with potential human health concerns and antibiotic resistance on children’s hands, instead of on the environmental sites, underscores the importance of good hand hygiene practices in kindergartens. Furthermore, the understanding that fomite surface, such as toys, carries high numbers of pathogens contamination should help to identify sites which represent a risk and facilitate risk management approach for indoor hygiene in kindergarten to minimize the spread of harmful microbes indoors. Based on these findings, large-scale studies that account for multiple locations, temporal aspects and occupancy factors should help to define a “baseline of healthy ecosystem” for kindergarten, and eventually lead to a rational strategy that formulates health-promoting symbiosis between young children and their indoor environments.

## Data Availability Statement

The datasets presented in this study can be found in online repositories. The names of the repository/repositories and accession number(s) can be found at: https://www.ebi.ac.uk/ena, PRJEB40975.

## Ethics Statement

The studies involving human participants were reviewed and approved by The Ethics Committee of Qingdao Institute of BioEnergy and Bioprocess Technology, Chinese Academy of Sciences. Written informed consent to participate in this study was provided by the participants’ legal guardian/next of kin.

## Author Contributions

TL, JX, PZ, JL, and HZ conceived and devised the overall research goals and aims. TL, JL, PZ, and LZ planned and executed the study implementation. PZ, LZ, and YD contributed to the sampling processing and performed the laboratory experiments. TL and DC processed the experimental data and performed the analysis and interpretation of the results. TL, DC, JX, and JL wrote the manuscript in consultation with PZ, LZ, YD, and HZ. JX, JL, and TL provided the overall project supervision. All authors contributed to the article and approved the submitted version.

## Funding

This work was supported by P&G-CAS iMicrobiome Collaborative Initiative, Shenzhen Province Research Fund (SGDX2019081623060946) and Shandong Energy Institute.

## Conflict of Interest

TL, DC, JL, and HZ are employed by company Procter & Gamble.

The remaining authors declare that the research was conducted in the absence of any commercial or financial relationships that could be construed as a potential conflict of interest.

## Publisher’s Note

All claims expressed in this article are solely those of the authors and do not necessarily represent those of their affiliated organizations, or those of the publisher, the editors and the reviewers. Any product that may be evaluated in this article, or claim that may be made by its manufacturer, is not guaranteed or endorsed by the publisher.
