# Supplementary material for: Species-Resolved Metagenomics of Kindergarten Microbiomes Reveal Microbial Admixture Within Sites and Potential Microbial Hazards
Source: Front Microbiol. 2022 Mar 28;13:871017. doi: 10.3389/fmicb.2022.871017 (PMC8996153; doi:10.3389/fmicb.2022.871017)
Supplement: Supplementary file 1 [file Data_Sheet_1.docx]

**Supplementary Materials for**

**Species-resolved metagenomics of kindergarten microbiomes reveal microbial admixture within sites and potential microbial hazards**

Tze Hau Lam^1^, Dillon Chew^1^, Helen Zhao^1^, Pengfei Zhu^2^, Lili Zhang^2^, Yajie Dai^2^, Jiquan, Liu^1#^, Jian Xu^2, 3#^

^1^Procter & Gamble Singapore Innovation Center, Singapore

^2^Single-Cell Center, CAS Key Laboratory of Biofuels and Shandong Key Laboratory of Energy Genetics, Shandong Energy Institute, Qingdao Institute of BioEnergy and Bioprocess Technology, Chinese Academy of Sciences, Qingdao, Shandong, China

^3^University of Chinese Academy of Sciences, Beijing, China

**SUPPLEMENTAL TABLES AND FIGURES**

**Supplementary Table 1. Validation of the 2bRAD-M microbiome sequencing method using a mock microbiota consisting of 20 bacterial species in equal abundance (MSA-1002).**

| **Species** | **Rel. Abundance**  **(2b-RAD-M)** | **Rel. Abundance**  **(MSA-1002)** |
| --- | --- | --- |
| *Acinetobacter baumannii* | 0.0863 | 0.05 |
| *Bacillus cereus* | 0.0265 | 0.05 |
| *Bacteroides vulgatus* | 0.0808 | 0.05 |
| *Bifidobacterium adolescentis* | 0.0428 | 0.05 |
| *Clostridium beijerinckii* | 0.0339 | 0.05 |
| *Cutibacterium acnes* | 0.0534 | 0.05 |
| *Deinococcus radiodurans* | 0.0380 | 0.05 |
| *Enterococcus faecalis* | 0.0565 | 0.05 |
| *Escherichia coli* | 0.0485 | 0.05 |
| *Helicobacter pylori* | 0.0626 | 0.05 |
| *Lactobacillus gasseri* | 0.0721 | 0.05 |
| *Neisseria meningitidis* | 0.0582 | 0.05 |
| *Porphyromonas gingivalis* | 0.0709 | 0.05 |
| *Pseudomonas aeruginosa* | 0.0249 | 0.05 |
| *Rhodobacter sphaeroides* | 0.0295 | 0.05 |
| *Schaalia odontolytica* | 0.0274 | 0.05 |
| *Staphylococcus aureus* | 0.0876 | 0.05 |
| *Staphylococcus epidermidis* | 0.0526 | 0.05 |
| *Streptococcus agalactiae* | 0.00 | 0.05 |
| *Streptococcus mutans* | 0.0473 | 0.05 |


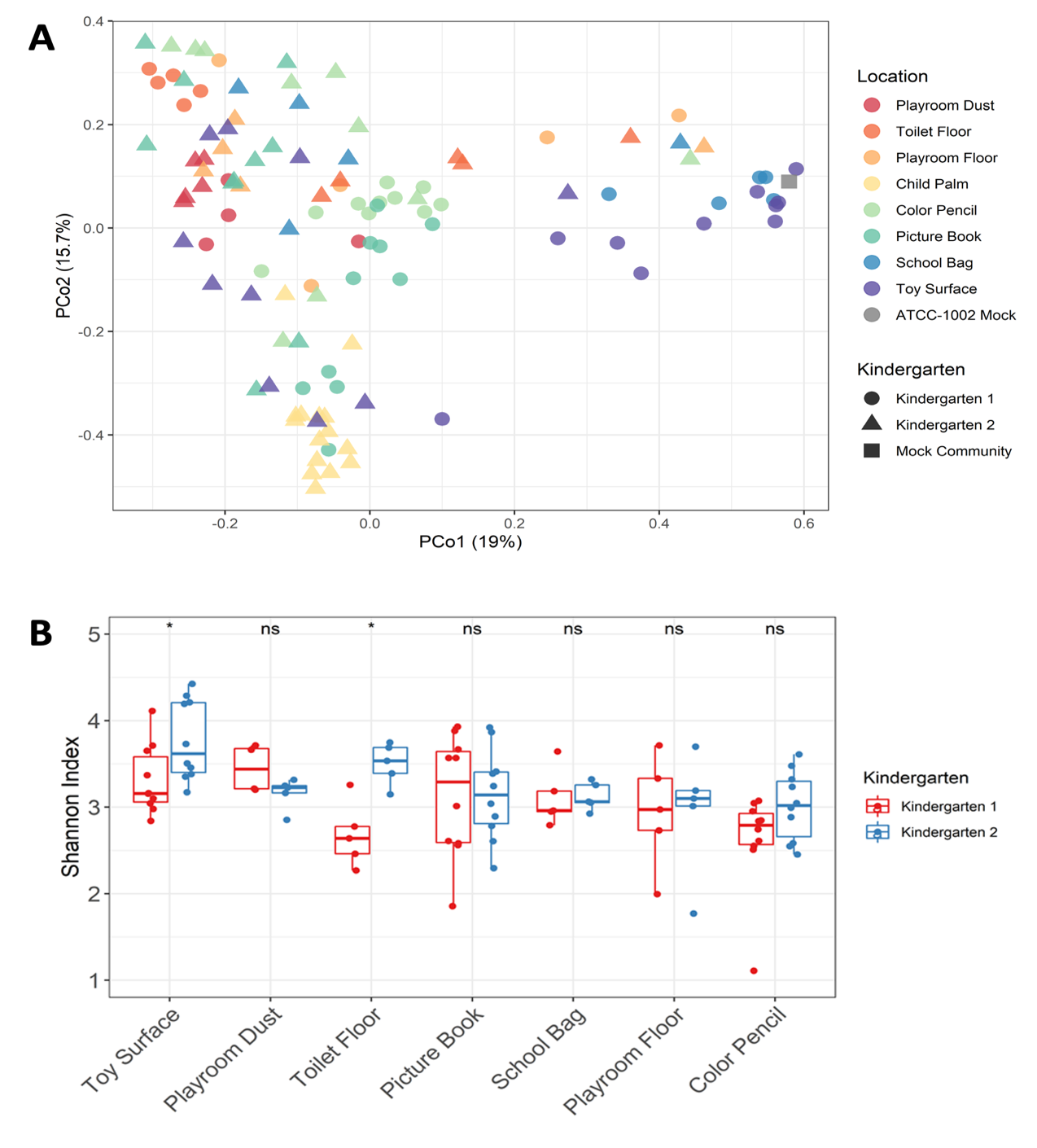


**Supplementary Figure 1. Comparison of microbial communities between samples.** The Principal coordinates analysis (PCoA) ordination plot based on Bray-Curtis distance where each point corresponds to a sample, is colored according to the sampled location, and has a shape based on which kindergarten the sample was obtained from.

**
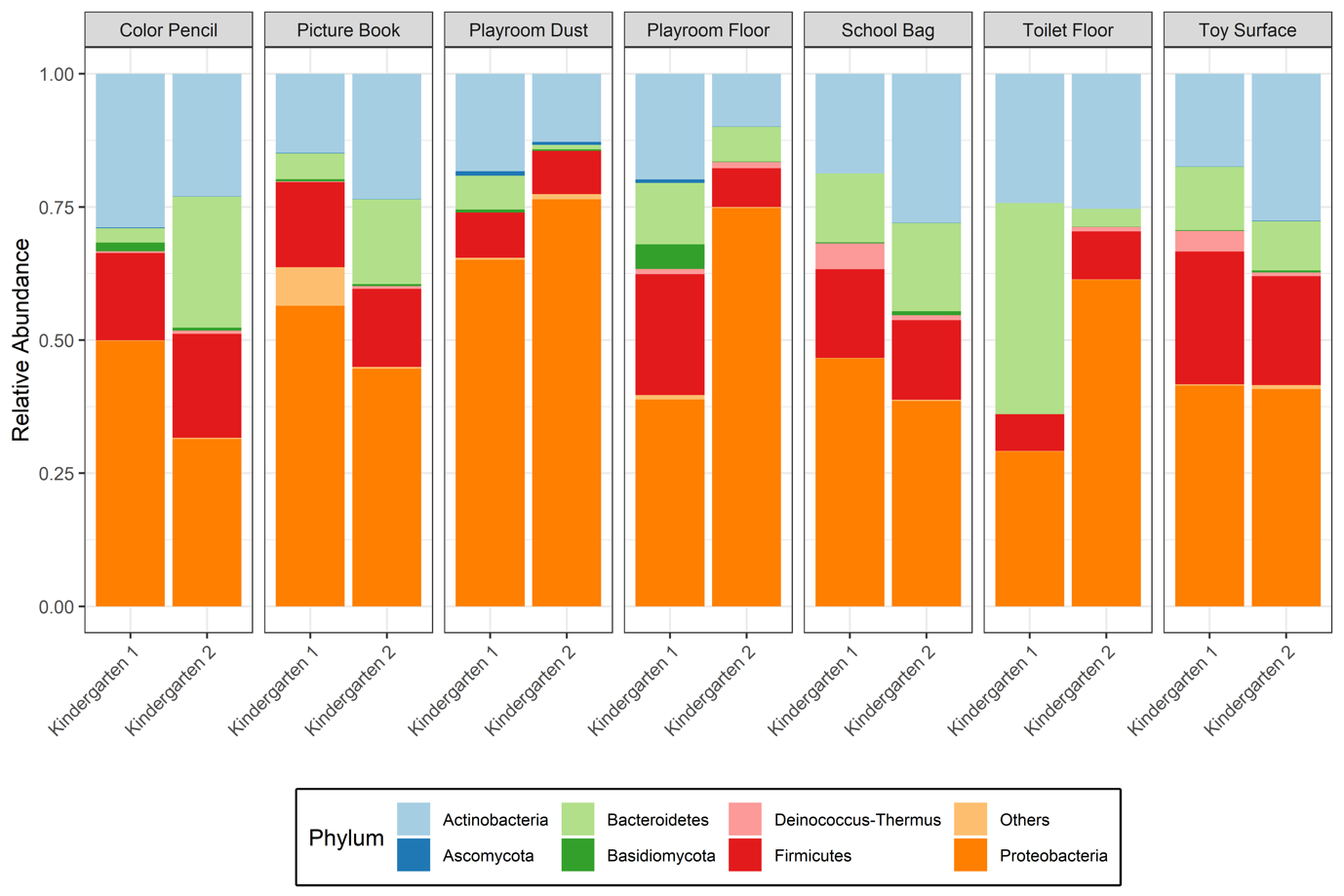
**

**Supplementary Figure 2. A Phylum-level overview of the microbial communities in both kindergartens.** Relative abundance of the different phyla across all sites in both kindergartens are presented. Those phyla with mean relative abundance of < 0.001 are grouped under “Others”.

**
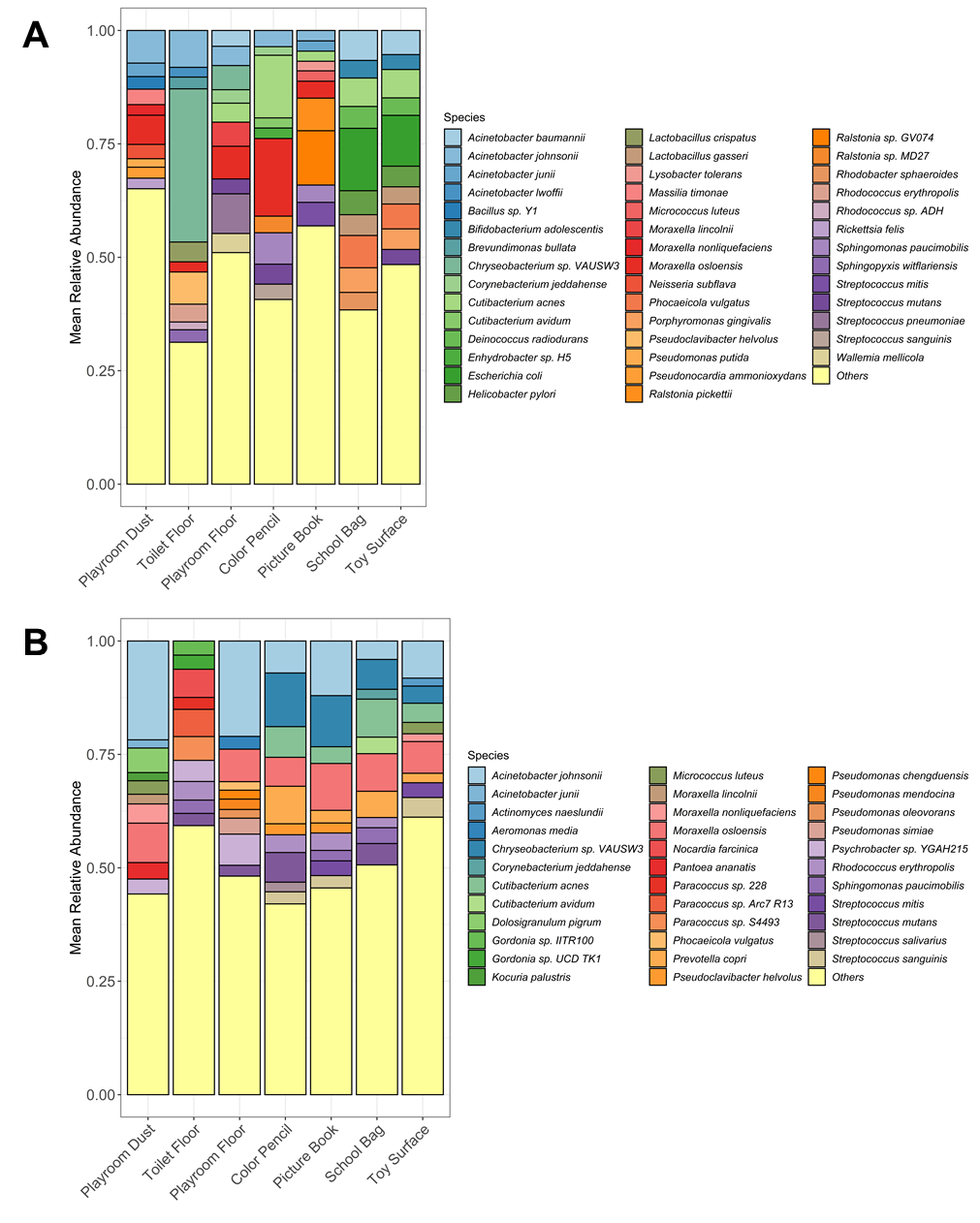
Supplementary Figure 3. The Species-level composition of the microbial communities across sampled sites in both kindergartens.** Relative abundance of the top 10 species (ranked via mean relative abundance) across all sites in both Kindergarten (**A)** 1 and (**B)** 2 are presented while the rest are grouped under “Others”.


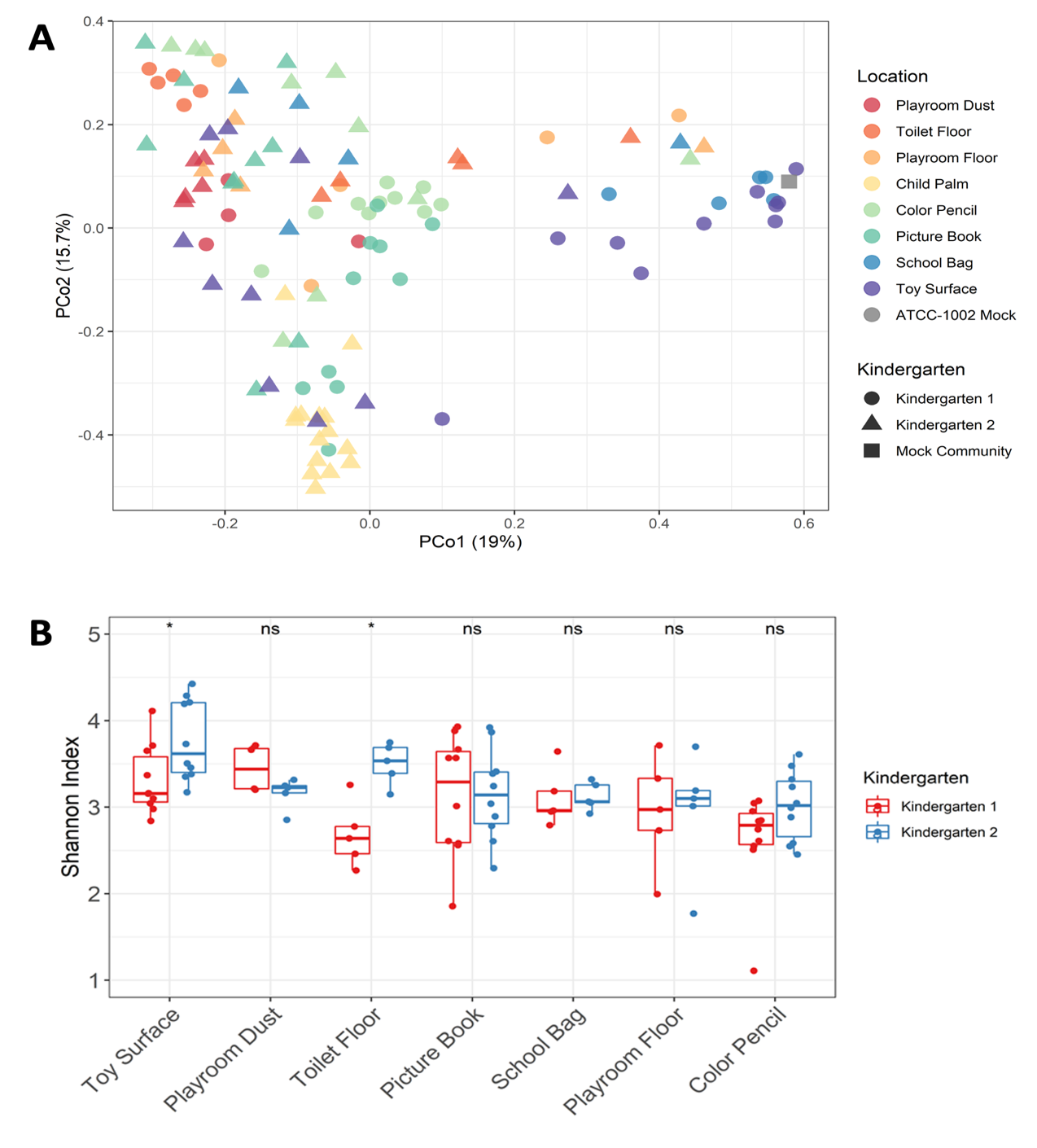
**Supplementary Figure 4. The Species-level diversity of the microbial communities of both kindergartens.** The Shannon diversity was observed to differ significantly between the two kindergartens at the Toy Surface (*P* = 0.015) and the Toilet Floor (*P* = 0.016). The *P* values shown above the boxplots were derived using the Mann-Whitney *U* test: *ns*: not significant, *: *P* ≤ 0.05, **: *P* ≤ 0.01.


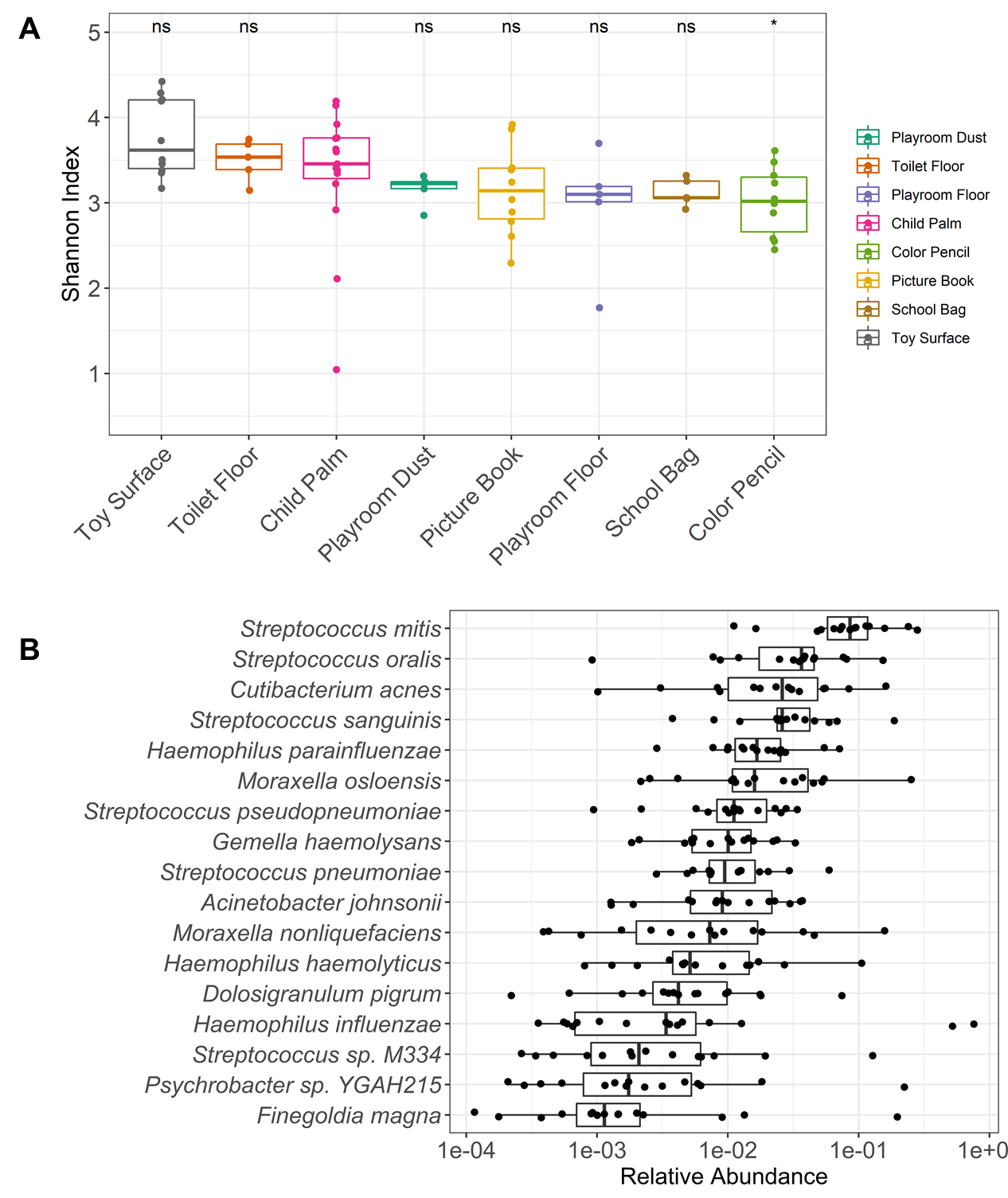


**Supplementary Figure 5. The Species-level diversity and composition of the microbial communities present on Child Palm**. **(A)** With Child Palm as the reference group, the Shannon diversity was observed to only differ significantly with Color Pencil (*P* = 0.048). The *P* values shown above the boxplots were derived using the Mann-Whitney *U* test: *ns*: not significant, *: *P* ≤ 0.05, **: *P* ≤ 0.01. **(B)** Samples (n=15) were obtained from children in Kindergarten 2 and only species with mean relative abundance > 1% are shown in the figure.
